# A re-assessment of 4CMenB vaccine effectiveness against serogroup B invasive meningococcal disease in England based on an incidence model

**DOI:** 10.1186/s12879-021-06906-x

**Published:** 2021-12-11

**Authors:** Lorenzo Argante, Victoria Abbing-Karahagopian, Kumaran Vadivelu, Rino Rappuoli, Duccio Medini

**Affiliations:** 1 GSK, Siena, Italy; 2 GSK, Amsterdam, The Netherlands

**Keywords:** Invasive meningococcal disease, IMD, MenB, 4CMenB, Bexsero, Effectiveness, VE, Incidence model, Screening method, Epidemiology

## Abstract

**Background:**

The four-component serogroup B meningococcal 4CMenB vaccine (*Bexsero*, GSK) has been routinely given to all infants in the United Kingdom at 2, 4 and 12 months of age since September 2015. After 3 years, Public Health England (PHE) reported a 75% [95% confidence interval 64%; 81%] reduction in the incidence of serogroup B invasive meningococcal disease (IMD) in age groups eligible to be fully vaccinated. In contrast, vaccine effectiveness (VE) evaluated in the same immunization program applying the screening method was not statistically significant. We re-analyzed the data using an incidence model.

**Methods:**

Aggregate data—stratified by age, year and doses received—were provided by PHE: serogroup B IMD case counts for the entire population of England (years 2011–2018) and 4CMenB vaccine uptake in infants. We combined uptake with national population estimates to obtain counts of vaccinated and unvaccinated person-time by age and time. We re-estimated VE comparing incidence rates in vaccinated and non-vaccinated subjects using a Bayesian Poisson model for case counts with person-time data as an offset. The model was adjusted for age, time and number of doses received.

**Results:**

The incidence model showed that cases decreased until 2013–2014, followed by an increasing trend that continued in the non-vaccinated population during the immunization program. VE in fully vaccinated subjects (three doses) was 80.1% [95% Bayesian credible interval (BCI): 70.3%; 86.7%]. After a single dose, VE was 33.5% [12.4%; 49.7%]_95%BCI_ and after two doses, 78.7% [71.5%; 84.5%]_95%BCI_. We estimated that vaccination averted 312 cases [252; 368]_95%BCI_ between 2015 and 2018. VE was in line with the previously reported incidence reduction.

**Conclusions:**

Our estimates of VE had higher precision than previous estimates based on the screening method, which were statistically not significant, and in line with the 75% incidence reduction previously reported by PHE. When disease incidence is low and vaccine uptake is high, the screening method applied to cases exclusively from the population eligible for vaccination may not be precise enough and may produce misleading point-estimates. Precise and accurate VE estimates are fundamental to inform public health decision making. VE assessment can be enhanced using models that leverage data on subjects not eligible for vaccination.

**Supplementary Information:**

The online version contains supplementary material available at 10.1186/s12879-021-06906-x.

## Background

*Neisseria meningitidis* is a strictly human bacterium that can cause invasive meningococcal disease (IMD). The most frequent clinical manifestations of IMD are meningitis and sepsis, both serious and rapidly fulminant conditions associated with considerable mortality and sequelae worldwide [1–3]. Six serogroups (A, B, C, W, X, and Y) are responsible for most IMD cases, and the disease incidence is generally highest in infants, showing geographically asynchronous secular trends leading to substantial temporal variability in the number of cases [2, 4]. Serogroup B is currently a major cause of IMD in the Americas, Australia and Europe [5].

Pre-licensure efficacy trials for meningococcal vaccines are not feasible because the incidence of IMD is low. Hence, licensure of the four-component serogroup B meningococcal 4CMenB vaccine (*Bexsero*, GSK) was based on studies testing its safety and immunogenicity, which is measured through a serum bactericidal assay with human complement that correlates with protection [6, 7]. Accordingly, real-world studies of vaccine effectiveness (VE) and impact are deemed very important.

The first such real-world study looking into 4CMenB vaccine effect was conducted by Public Health England (PHE). In September 2015, the United Kingdom became the first country to include 4CMenB in its national immunization program offering a reduced schedule of three doses of 4CMenB to all infants: two primary doses at 2 and 4 months of age and a booster at age 12 months [8]. After 3 years of 4CMenB vaccination, PHE reported a very high vaccine uptake (92.5% for the primary two-dose immunization and 87.9% for the third dose) and a statistically significant 75% reduction of the incidence of serogroup B IMD (95% confidence interval [CI] 64%; 81%). This vaccine impact (VI) was estimated on age groups fully eligible for vaccination (that also included non-vaccinated and partially vaccinated subjects) through a Poisson model [9].

In contrast, the same study reported VE estimates that were lower than the VI and not significantly different from zero (e.g., in subjects fully vaccinated with three doses, 59.1% [− 31.1%; 87.2%]_95%CI_) [9]. VE was estimated using the screening method, an approach that expresses the effectiveness as a function of the proportion of cases that has been vaccinated and the vaccine uptake in the population [10–12]. VE was estimated from fully vaccinated cases compared with non-vaccinated cases eligible for three doses. VI was estimated from a population that included the same cases used for the VE, with in addition, other partially and non-vaccinated subjects and cases. Therefore, theoretically, VI should not exceed the VE in fully vaccinated. Indeed, when indirect effects of a vaccine are absent or negligible, especially at the beginning of an immunization program, VI should theoretically equal its effectiveness multiplied by the proportion of vaccinated persons (i.e., the vaccine uptake $x$): $VI=VE*x$. This is a simple mathematical relation that is valid in the absence of trends in disease incidence and vaccine uptake but can give useful indications for VI and has been previously used to broadly estimate VE from impact and uptake [13]. We provide its formal derivation and additional details on underlying assumptions in Additional file 1: Section S1. Since $x$ cannot be higher than one (i.e., 100% uptake), it follows that VE should be at least as high as VI. Although the measured 75% impact is reassuring of the fact that the immunization program with 4CMenB in England has been successfull, the undefined effectiveness (not significantly greater than zero) does not allow comparing the effectiveness of complete or incomplete vaccination and drawing conclusions about the applied vaccine schedule. Moreover, definite and precise VE estimates are necessary to predict the impact of a similar immunization program in other countries and to parametrize cost-effectiveness models.

The lack of significance for VE estimates warrants a rigorous re-estimation of 4CMenB effectiveness with greater precision. We analyzed real-world data on serogroup B IMD and 4CMenB uptake between September 2011 and August 2018 provided to us by PHE and re-assessed the effectiveness of one to three doses of 4CMenB using an incidence model.

## Methods

### Disease cases and vaccine uptake

All the data on IMD and vaccine uptake were provided to us by PHE. Detailed information on the implementation of the immunization program, on PHE’s routine surveillance of patients with IMD in England and on vaccine uptake data collection are available in the original publication by Ladhani and co-authors [9].

The received data on IMD (Additional file 1: Table S1) were case counts of laboratory-confirmed serogroup B IMD cases from the entire population of England, aggregated by age (11 age groups; 0–1 month of age to older than 44 years of age), by year of disease onset and by number of 4CMenB doses received at least 14 days before disease onset (14 days is the time to mount an immune response). The time period spanned 7 years, from September 2011 through August 2018. Time units were years (from September through the following August). 4CMenB vaccination started in September 2015; therefore, data covered four pre-vaccination years and 3 years during the immunization program. The number of doses received ranged from zero, (unvaccinated), to three (fully vaccinated). Before September 2015 all the cases were unvaccinated.

The data on vaccine uptake provided by PHE relied on two different data sources: (i) the proportions of all the infants eligible to the immunization program that received one to three doses of 4CMenB at 6, 12 and 18 months of age, by month of time; (ii) the same proportions by day of age and day of time for nearly 60,000 individuals (30,000 for the third dose) from different geographical areas across England. The first data source was relative to the entire eligible population in England; the second had a finer time step and, after being rescaled to fit the first data source, was used to calculate uptake at ages different than 6, 12 and 18 months (we followed the same approach used in the original study by Ladhani and co-authors [9], as described in Additional file 1: Section S3).

In addition to the above-described data, we used population estimates for England (years 2011–2018) from the Office for National Statistics of the United Kingdom (publicly available at https://www.ons.gov.uk). We grouped the population estimates in the same age-and-time structure as the IMD case counts. Then, we combined population estimates with the estimated uptake proportions to derive the number of person-years for each age group defined by PHE, for each year (from September through August) and for each number of doses received at least 14 days earlier. Details are provided in Additional file 1: Section S3 and person-years are plotted in Additional file 1: Figures S1 and S2.

### Model-based VE re-assessment

The effectiveness of 4CMenB was re-evaluated comparing serogroup B IMD incidence rates $IR$ in vaccinated with $IR$ in non-vaccinated subjects [12]:

1 $$VE= \frac{{IR}_{\text{unvaccinated}}-{IR}_{\text{vaccinated}}}{{IR}_{\text{unvaccinated}}}=1-IRR,$$

where $IRR$ is an incidence rate ratio.

Since the data were counts, we used a Poisson model (here sometimes called P1 model when compared to alternative models) [14]. We employed a Bayesian approach [15, 16]. Bayesian methods have already been used to estimate VE [17]. Specifically, we modelled counts of serogroup B IMD cases ${y}_{a,t,d}$ with a hierarchical Poisson regression model [15], adjusting for age $a$, year $t$ and number of vaccine doses $d$, using person-time ${N}_{a,t,d}$ as an offset:

2 $$\begin{gathered} y_{a,t,d} \sim {\text{Poisson}} \left( {N_{a,t,d} e^{{\rho_{a} + \beta_{t} + \theta_{d} }} } \right) , \hfill \\ \beta_{t} \sim {\text{Normal}} \left( {0; \sigma_{\beta }^{2} } \right) , \hfill \\ \end{gathered}$$

where ${e}^{{\rho }_{a}}$ is the average incidence rate in the unvaccinated population in age group $a$. The parameter ${\beta }_{t}$ adjusts for time, while ${\theta }_{d}$ controls for the number of doses $d$ received at least 2 weeks before. ${\beta }_{t}$ was constrained to zero sum: their values must be considered relative to the average $\overline{\beta }$ across the full study period, i.e. $\overline{\beta }$ is the baseline for ${\beta }_{t}$.

With this parametrization, ${e}^{{\rho }_{a}+{\beta }_{t}}$ is the incidence rate in unvaccinated subjects, corrected for age and year. We fixed ${\theta }_{0}=0$ as a baseline for $\theta$, so that ${e}^{{\theta }_{d}}$ is the age-and-time-adjusted incidence rate ratio ${IRR}_{d}$ in vaccinated subjects that received $d>0$ doses relative to unvaccinated subjects. Therefore, consistent with the VE definition, the effectiveness of $d$ doses was:

3 $${{VE}_{d}=1-{IRR}_{d}=1-e}^{{\theta }_{d}} .$$

Since we fixed ${\theta }_{0}=0$, it follows that ${VE}_{0}=0$, meaning that the vaccine was assumed to have no effect on unvaccinated groups (no indirect effects), i.e. when $d=0$. We assumed no indirect effects due to herd immunity, as none has been previously observed [9]. Anyway, it is reasonable to neglect possible indirect effects, since no more than 2% of England’s population could have been fully vaccinated during the first 3 years of program, probably not enough to see herd immunity effects at population level. We assigned non-informative prior distributions to all the model’s parameters and numerically estimated the respective posterior distributions, given prior, data and model. We chose non-informative priors so that posterior values are affected only by data and model through the assumed Poisson likelihood.

Bayesian estimation was numerically executed using Markov chain Monte Carlo. Specifically, we used a Hamiltonian Monte Carlo algorithm: the No-U-Turn sampler of Python’s PyMC3 package (further details in Additional file 1: Section S4) [18]. Posterior distributions of the parameters were summarized through their point estimates (posterior means) and the limits of their 95% Bayesian credible interval (95%BCI, calculated as the 95% highest posterior density). VEs were derived from $\theta$ through Eq. 3.

The incidence model that we used (Eq. 2) is based on the assumption that for non-vaccinated individuals, relative changes in incidence over time are equal for different age groups. In other words, we did not use interaction terms between age and time. This assumption was carefully evaluated comparing fits with the data, as shown in the second section of the results in this manuscript. Moreover, drastic changes in meningococcal incidence for specific age groups with respect to other are unusual unless related to the emergence of large outbreaks. To our knowledge, there were no outbreaks in England in any age group during the study period, only sporadic cases.

### Model selection and diagnostics

In addition to the P1 model (Eq. 2) we tested nine other models: P2, P3, P4, P5, NB1, NB2, NB3, NB4 and NB5. NB1 was the same model as P1 with a negative binomial likelihood instead of a Poisson likelihood, to account for possible overdispersion. P2 and NB2 were the same as P1 and NB1, respectively, but non-hierarchical (i.e., ${\beta }_{t}$ parameters were not pooled from a normal distribution; each ${\beta }_{t}$ was estimated independently). Models P3 and NB3 were, respectively, derived from P1 and NB1 by fixing ${\beta }_{t}$ to zero, i.e., without adjusting for variations in incidence over time. P4 and P5 were, respectively, a hierarchical and a non-hierarchical model, in which (compared to P1) ${\rho }_{a}+{\beta }_{t}$ were replaced with a single parameter ${\gamma }_{at}$ that depends on both age and time, thus relaxing the assumption of independence between age and time, while accounting for a greater number of parameters. Finally, NB4 and NB5 were similar to P4 and P5 with a negative binomial instead of a Poisson likelihood, to account for overdispersion. All these models are reported in detail in Additional file 1: Section S7.

For each model we first evaluated Monte Carlo convergence, by visually controlling chains and by calculating $\widehat{\text{R}}$ statistics for each parameter of each model. $\widehat{\text{R}}$ is the factor by which the scale of the distribution for a sampled parameter might be reduced if the simulations were continued in the limit n → ∞ [15, 19]. Values near one indicate convergence, i.e., parameter space has been optimally explored. Models’ goodness of fit were compared and ranked using two different deviance criteria to measure relative goodness of fit: the leave-one-out cross-validation (LOO-CV) and the widely applicable information criterion (WAIC) [15]. Both WAIC and LOO-CV account for overfitting by penalizing complex models, with lower values corresponding to better fits. $\widehat{\text{R}}$, WAIC and LOO-CV were calculated through Python’s PyMC3 package [18].

### Expected cases, counterfactual and averted cases

To estimate the number of averted cases we used the model to generate a counterfactual (i.e., the expected case counts if no immunization program was implemented) and compared it with the expected case counts from the model’s best fit on the observed data. In practice, posterior distributions of expected cases were numerically generated by randomly sampling from the fitted model (100,000 iterations). Counterfactuals were sampled in the same manner, except that all the effectiveness parameters ($\theta$ in the model) were fixed to zero, to simulate the absence of vaccination. Probability distributions of the averted cases imputable to the vaccine were calculated by subtracting counterfactuals from best-fitted expected cases. All the distributions were summarized and reported as mean and 95%BCI. Further details are reported in Additional file 1: Section S5.

### Expected VE using the mathematical relation between impact, uptake and VE

When indirect effects are absent or negligible, the impact of a vaccine equals VE multiplied by the vaccine uptake [13]. For a three-dose vaccine, the formula becomes (full derivation in Additional file 1: Section S1):

4 $$VI={x}_{1}{VE}_{1}+{x}_{2}{VE}_{2}+{x}_{3}{VE}_{3 },$$

where $VI$ is the vaccine impact and ${x}_{1}$, ${x}_{2}$ and ${x}_{3}$ are the proportions of subject vaccinated with one to three doses.

## Results

### Estimates of incidence rates and VE

The re-analysis of data on IMD cases and 4CMenB uptake using an incidence model allowed us to obtain point estimates and 95% BCIs for four relevant measures: (i) average IMD incidence rates per age group in non-vaccinated subjects in the period from September 2011 through August 2018, (ii) the variation of incidence rates over time in non-vaccinated subjects relative to their baseline, (iii) incidence rate ratios in vaccinated with respect to non-vaccinated subjects and (iv) VE of 4CMenB. Additional file 1: Table S2 reports the list of all the fitted parameters.

**Fig. 1:**
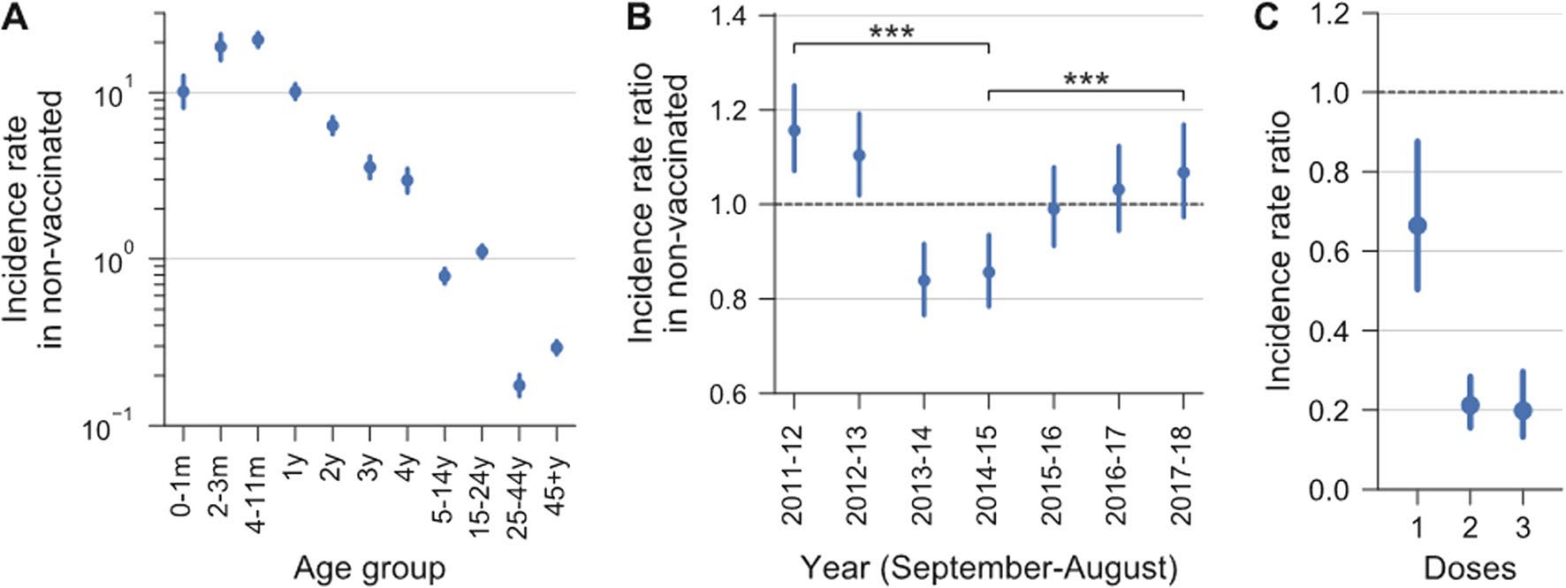
Incidence rates. **A** Incidence rates by age in the non-vaccinated population, averaged over the period from September 2011 to August 2018 (cases per 100,000 person-years, ${\mathrm{e}}^{{\uprho }_{\mathrm{a}}}$ in the model; m: months, y: years). **B** Are yearly incidence rates ratios (${\mathrm{e}}^{{\upbeta }_{\mathrm{t}}}$ in the model) in the non-vaccinated population (for the 4 years before immunization, i.e., from 2011 through 2015, this is the whole population), calculated relative to the 2011–2018 average (used as a baseline, fixed to 1, shown as a dashed horizontal line). **C** Incidence rate ratios in vaccinated relative to non-vaccinated subjects, by number of doses received (${e}^{{\theta }_{d}}$ in the model). Bars report 95% credible intervals around point estimates. P values: ***P < 0.001

Figure 1A shows that serogroup B IMD incidence rates per age group in the non-vaccinated population (2011–2018 average) were in line with previous observations [20]: the highest incidence was in infants (especially at 4–11 months of age); a relative maximum was observed in the 15–24 years age group; an increase was found in the last age group, which included the elderly.

Incidence rates in the non-vaccinated population varied significantly across years compared to the 2011–2018 average, exhibiting a decrease followed by an increasing trend (Fig. 1B). The incidence was highest during the first year of the study period (2011–2012, 1.16 times the average). Then it declined and reached its minimum in 2013–2014 (0.84 times the average). Thereafter, before and during the immunization program, the relative incidence in the non-vaccinated population continuously increased, up to 1.07 times the average in 2017–2018, which was 1.25 times significantly higher ([1.09; 1.43]_95%BCI_, P < 0.001) than the incidence observed during the last pre-vaccination year, i.e., 2014–2015.

**Fig. 2:**
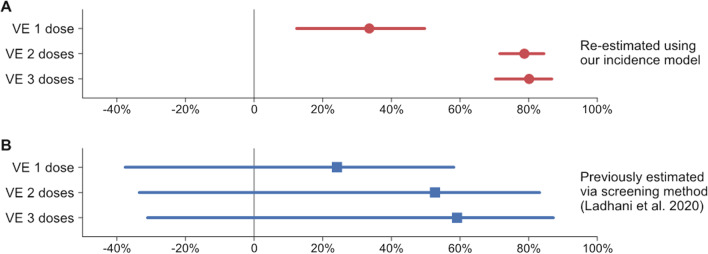
Re-assessed 4CMenB effectiveness compared with previous estimates. **A** In red: vaccine effectiveness (VE) of 4CMenB after one to three doses, re-estimated from data reported by Public Health England (PHE) using our Poisson regression model (Eq. 1). **B** In blue: VE estimated by PHE using the screening method [9]. Bayesian point estimates and 95% credible intervals are marked with red circles and bars. Frequentist point estimates and 95% confidence intervals are marked with blue squares and bars

Figure 1C shows the incidence rate ratios obtained from the best fit of the model to the data. Our estimates of the effectiveness of 4CMenB (Fig. 2A) show that VE was significantly higher than zero (P_VE ≤ 0_ = 0.0014) already after a single dose: 33.5%, [12.4%; 49.7%]_95%BCI_. After two doses, VE was 78.7% [71.5%; 84.5%]_95%BCI_ (P < 0.001). VE after the full three-dose schedule was 80.1%, [70.3%; 86.7%]_95%BCI_ (P < 0.001). Subjects who only received the first dose were nearly three times more at risk of disease than those who received two doses (incidence rate ratio: 3.13, [2.11; 4.62]_95%BCI_). For comparison, Fig. 2B shows the VE values estimated using the screening method in the original study [9].

### The fitted model accurately reproduced observed IMD case counts

**Fig. 3:**
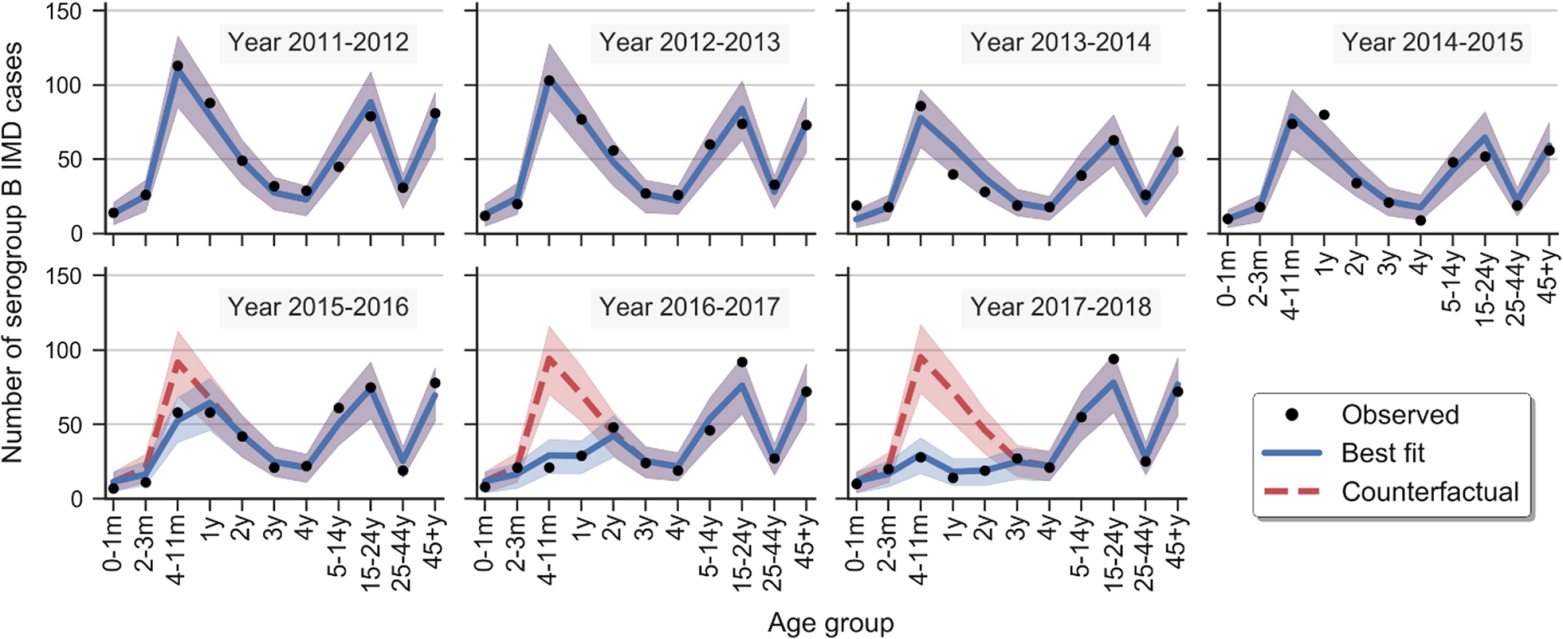
Observed, best fitted and counterfactual IMD case counts. Each plot reports as black points the yearly number of observed serogroup B invasive meningococcal disease (IMD) cases by age group (the first and second row report cases before and during immunization, respectively). Incidence model’s best fitted case counts are shown as blue solid lines. Red dashed lines report counterfactual IMD cases, expected in the absence of vaccination, generated through the model when setting VE to zero while leaving the other fitted parameters untouched. The blue and red semi-transparent regions are the 95% credible intervals of the corresponding expected case counts. m: age in months; y: age in years

Figure 3 shows the number of serogroup B IMD cases per year and age group, reported in England between September 2011 and August 2018, along with the best fit of the incidence model to the data (blue curves). Additional file 1: Figure S3 reports the same data and expected cases from the model’s fit also stratified by the number of 4CMenB doses received. The model accurately reproduced disease cases in each year, for every age group and number of doses received (Additional file 1: Figure S4 and Section S6, R^2^ predicted vs*.* observed > 0.96).

We tested alternative models and found four equally best-fitting models (Table 1): P1 (the Poisson model described here with Eq. 2), NB1 (the same as P1 but with a negative binomial instead of a Poisson counting process to account for possible overdispersion), P2 and NB2 (the same as P1 and NB1, respectively, but non-hierarchical, as described in detail in Additional file 1: Section S7). For these four models, deviance and estimated VEs were almost coincident. Therefore, we selected P1 as the best model because it is simpler than the other three. The other six tested models had poorer performances, as shown in Table 1. For models P3 and NB3 (derived from P1 and NB1 by fixing ${\beta }_{t}$ to zero, i.e., without adjusting for time), goodness of fit was significantly worse. The fit was also poorer when rejecting the assumption of independence between age and time parameters, i.e., by replacing ${\rho }_{a}+{\beta }_{t}$ with a single parameter ${\gamma }_{at}$ that depends on both age and time.

**Table 1:** Comparison between candidate models

Model	Convergence diagnostics	Goodness of fit (deviance) [SE]		Vaccine effectiveness (%) [95% BCI]		
	Maximum $\widehat{\text{R}}$	LOO-CV	WAIC	1 dose	2 doses	3 doses
NB1	1.00101	**622.4** [28.8]	**619.6** [28.4]	33.7 [13.2; 50.4]	78.7 [71.3; 84.3]	80.1 [70.4; 86.6]
P1	1.00004	**622.6** [28.8]	**619.9** [28.4]	33.5 [12.4; 49.7]	78.7 [71.5; 84.5]	80.1 [70.3; 86.7]
P2	1.00003	**622.9** [29.1]	**620.2** [28.7]	34.1 [13.4; 50.4]	78.9 [71.6; 84.4]	80.4 [70.5; 86.9]
NB2	1.00032	**623.1** [29.1]	**620.3** [28.7]	33.9 [12.7; 49.8]	78.9 [71.7; 84.5]	80.4 [70.7; 87.0]
NB3	1.00008	646.2 [27.0]	645.1 [26.9]	31.9 [7.7; 49.9]	76.0 [66.2; 83.2]	78.5 [66.7; 86.2]
P3	1.00005	671.3 [35.3]	669.7 [35.2]	31.1 [10.0; 47.3]	77.8 [70.5; 83.5]	78.9 [68.8; 85.9]
NB4	1.00052	675 [26.9]	633.1 [23.7]	21.1 [− 6.8; 41.7]	72.0 [60.7; 80.5]	76.2 [62.7; 85.2]
P4	1.00012	676.6 [27]	633 [23.7]	21.0 [− 6.6; 42.3]	71.9 [59.8; 80.2]	76.2 [62.2; 85.0]
P5	1.00011	706.3 [25.9]	641.8 [22.9]	25.6 [− 6.0; 47.7]	72.3 [58.8; 81.8]	75.0 [58.3; 85.4]
NB5	1.00038	707.1 [26]	642 [22.9]	25.8 [− 5.5; 48.6]	72.4 [58.2; 81.3]	74.9 [57.3; 85.2]

For each candidate model of the first column, the second column reports the maximum model’s $\widehat{\text{R}}$ factor as a diagnostic criterion of Monte Carlo convergence (values near one indicate that convergence was reached for all the models). Columns three and four report goodness of fits, calculated using the leave-one-out cross-validation (LOO-CV) and the widely applicable information criterion (WAIC), here reported with their standard errors (SE). The last three columns report final estimates of VE after one to three doses using the different models. The models are ordered by their LOO-CV score. Lowest deviance values (in bold) highlight four equivalently best-fitting models, from which we selected P1 as the best (Eq. 2 in the main text), the simplest of the four

### Counterfactual estimates and averted cases

Counterfactuals—i.e., expected numbers of serogroup B IMD cases that would have occurred in England if 4CMenB was not included in the national immunization program—are shown in Fig. 3 and Additional file 1: Figure S3 as red dashed curves. We calculated an excess of 312 [252; 368]_95%BCI_ cases prevented by the campaign during its first 3 years. Specifically, 46 [30; 62] during its first year, 113 [84; 140] during the second and 153 [117; 186] during the third year.

### Expected VI given our estimates of VE

Vaccine uptake of one, two and three doses in the fully eligible age groups on which PHE measured the VI were, respectively, ${x}_{1}$ = 3.6%, ${x}_{2}$ = 4.6%, ${x}_{3}$ = 87.9% [9]. We substituted these uptake values and our re-estimated VEs in the mathematical expression relating impact, uptake and VE for vaccinations with three doses (Eq. 4), and found an expected impact VI_exp_ = 75.2% [65.5%; 81.9%]_95%BCI_.

## Discussion

In this re-assessment we showed that the effectiveness of 4CMenB vaccine was 80.1% [70.3%; 86.7%]_95%BCI_ in fully vaccinated infants during its first 3 years of implementation in a national immunization program in England. We demonstrated that our VE estimates are in line with the previously reported VI: a 75% [64%; 81%]_95%CI_ reduction of serogroup B IMD incidence in age groups fully eligible for vaccination [9].

The same study [9] reported non-statistically significant VE, with point estimates more than 15% lower than the VI estimate, even in fully vaccinated subjects. They were calculated using the screening method, a simple and rapid approach where VE is estimated from the proportion of cases that are vaccinated and the proportion of the population vaccinated [10–12]. The study was designed to estimate direct effects of the vaccine by including only cases from the vaccine-targeted population [9], i.e., it a type I design [12]. In our re-analysis, we used also cases from the non-eligible population, assuming that indirect effects to be negligible. In other words, our design falls between designs type I and type IIb, where the latter normally estimates direct and indirect effects comparing a vaccinated community with a separate unvaccinated community [12].

The lack of precision of the estimates based on the screening method compared to our re-assessment may be because the screening method relies exclusively on cases emerging from the population eligible for vaccination. When the disease incidence is low and the vaccine uptake is high—as is the case in this study—the number of unvaccinated cases in the eligible population may be very low or even null (depending on the observational time), thus inadequate to produce sufficiently precise VE estimates [21]. Instead, our incidence model used non-vaccinated cases from the eligible as well as from the non-eligible population to achieve higher precision, adopting the additional assumption that relative changes in incidence over time are the same in different age groups, an assumption that held for the data analyzed here.

Although all our point estimates for VE fall inside the respective 95% CIs obtained via the screening method, point estimates produced with the screening approach are consistently lower than our results. The reason for this difference may be another consequence of the small number of cases that already caused the lack of precision. Indeed, the screening method was applied by matching the proportion of vaccinated subjects to the observed cases, according to the number of doses received, age and year at disease onset. As the number of disease cases was extremely small compared to the population eligible to the campaign, the matched vaccine uptake may not have been representative of the vaccine uptake in the population, especially with both disease incidence and uptake per dose greatly varying with age, as observed in this immunization program. Therefore, the low number of cases and a possible unlucky uptake matching may have just for chance driven VE point estimates towards lower values. In theory, it could have also led to the opposite effect, i.e., an overestimated VE point estimate, depending on age and time at disease for the limited number of cases to which the uptake was matched. The screening method would have likely required more years of surveillance to have enough cases for a precise evaluation of the proportion of vaccinated population. Instead of only using uptake data relative to cases, we used more robust person-time data of the whole vaccinated and unvaccinated population (mostly composed of non-cases) and could estimate VE in line with the previously reported incidence reduction [9].

Most relevant from a public health perspective is the overall number of averted cases. We estimated this to be 312 [252–369]_95%BCI_, which is in good agreement with the 277 [236–323]_95%CI_ averted cases estimated by PHE [9]_._ Disease incidence decreased during the first two pre-vaccination years considered here, then slowly inverted its trend, started to rise and continuously increased—in non-vaccinated subjects—after the inclusion of 4CMenB in England’s infant national immunization program in September 2015 (Fig. 1, panel B). Thus the decision to include 4CMenB in the national immunization program was timely.

Although our VE estimates were more precise than those obtained with the screening method and agreed with the incidence reduction, our analysis has some limitations. The data on vaccination used in this re-assessment were originally collected to apply the screening method. Therefore, information on vaccination status at an individual level was collected for cases only. By design, the vaccination status of non-cases, i.e., controls, was derived from uptake statistics that came from a source external to the study [11]. Also, data on disease that we received were case counts, already aggregated by age, year and doses. Even if we believe that the stratification was optimal for this kind of analysis, we could not test the sensitivity of the results when varying the stratification. Another possible limitation is that we could not test covariates other than age, time and doses received, to find and adjust for potential additional confounders. An intrinsic weakness of our model-based approach is that it assumes incidence rates are constant within each group. Most importantly, we assumed that yearly incidence variation would affect each age group proportionally. In case of a drastic variation of the incidence in specific age groups, as it may happen in case of IMD outbreaks, this model could not hold. In that case, individuals living in geographical areas or specific settings (e.g., schools) particularly affected by outbreaks may be excluded from the analysis, or more complex transmission models to predict incidence trends would be needed. However, we have verified that our model accurately reproduced the data, indicating that the model was appropriate for this context. The approach that we adopted assumes that possible indirect effects due to herd immunity are absent or negligible (as previously observed for this study) [9], as the number of infants vaccinated up to August 2018 was low compared to the entire England’s population. Nevertheless, in case of vaccine-induced herd immunity, the incidence model and VE estimates could be affected by indirect effects.

Precise and accurate estimates of 4CMenB effectiveness are essential to appropriately inform cost-effectiveness analyses that support public health decision making [22, 23]. Provided the availability of strain typing data [24, 25] on the same disease cases used for this re-assessment, the higher precision of our approach would allow further stratifying data and to assessing 4CMenB effectiveness against different strain types. Such estimates, combined with strain typing data from other countries [26], would in turn allow even more robust predictions of 4CMenB effects in different geographies, improving cost-effectiveness evaluations. In the future, our approach may be adopted in similar settings by using surveillance data at a population level during vaccination programs, if more traditional methods are underpowered.

Our results are also aligned with 4CMenB effectiveness in infants and children reported in two recent observational studies in Italy (two regions: Tuscany and Veneto) and Portugal [27, 28]. VE estimates reported in these two studies, which were performed in settings with specifications different to England, confirm once again our re-assessment findings.

## Conclusions

Our re-analysis quantified the real-world effectiveness of 4CMenB during a national infant immunization program and confirmed evidence of protection against serogroup B invasive disease. The screening method may be inadequate in settings characterized by high vaccine uptake and low disease incidence, whereas carefully calibrated incidence models that more extensively and efficiently use the same kind of surveillance data may be more appropriate to assess effectiveness of meningococcal vaccines.

## Supplementary Information

**Additional file 1.** **Section S1.** Relationship between impact, uptake and effectiveness. **Section S2.** Case data. **Section S3.** Vaccine uptake and person-years data. **Section S4.** Model’s inference. **Section S5.** Model’s predictions. **Section S6.** Predictive accuracy of the incidence model. **Section S7.** Alternative models. **Table S1.** Serogroup B IMD case counts shared by PHE and used for our re-assessment. **Table S2.** Best estimates of the parameters. **Figure S1.** Person-years by age and year. **Figure S2.** Person-years / 100,000 by age, year and doses received. **Figure S3.** Case data and model predictions by age, time and doses received. **Figure S4.** Residuals and predicted vs. observed cases. **Figure S5.** Sampled parameters and their distributions.

## Data Availability

All data generated or analyzed during this study are included in this published article and its additional information files. The case counts of serogroup B IMD in England as used in this analysis (stratified by age, year and doses received) are provided as Additional file 1: Table S1.

## Abbreviations

VE: Vaccine effectiveness
VI: Vaccine impact
IMD: Invasive meningococcal disease
hSBA: Serum bactericidal assay with human complement
PHE: Public Health England
CI: Confidence interval
BCI: Bayesian credible interval
