# Supplementary material for: A re-assessment of 4CMenB vaccine effectiveness against serogroup B invasive meningococcal disease in England based on an incidence model
Source: BMC Infect Dis. 2021 Dec 11;21:1244. doi: 10.1186/s12879-021-06906-x (PMC8666080; doi:10.1186/s12879-021-06906-x)
Supplement: Supplementary file 1 — Additional file 1. Section S1. Relationship between impact, uptake and effectiveness. Section S2. Case data. Section S3. Vaccine uptake and person-years data. Section S4. Model’s inference. Section S5. Model’s predictions. Section S6. Predictive accuracy of the incidence model. Section S7. Alternative models. Table S1. Serogroup B IMD case counts shared by PHE and used for our re-assessment. Table S2. Best estimates of the parameters. Figure S1. Person-years by age and year. Figure S2. Person-years / 100,000 by age, year and doses received. Figure S3. Case data and model predictions by age, time and doses received. Figure S4. Residuals and predicted vs. observed cases. Figure S5. Sampled parameters and their distributions. [file 12879_2021_6906_MOESM1_ESM.docx]

A re-assessment of 4CMenB vaccine effectiveness against serogroup B meningococcal disease in England based on an incidence model

Supplementary information

Lorenzo Argante^1^, Victoria Abbing-Karahagopian^2^, Kumaran Vadivelu^1^, Rino Rappuoli^1^ and Duccio Medini^1^

*^1^GSK, Siena, Italy*

*^2^GSK, Amsterdam, the Netherlands*

# S1 Relationship between impact, uptake and effectiveness

The vaccine impact (VI) during a country-level immunization program depends on both direct and indirect effectiveness, and on the proportion of the considered population that has been vaccinated.^1^ When indirect effects (i.e., disease risk reduction in the whole population, including non-vaccinated, due to a vaccine-induced decrease in the pathogen’s transmissibility) are absent or negligible, especially at the beginning of the campaign, VI simply equals the vaccine effectiveness (VE) multiplied by the proportion of vaccinated persons (i.e., the vaccine uptake). This simple mathematical relationship has already been used to evaluate the possible effectiveness of a vaccine for which impact and uptake are known.^2^ We provide here its formal derivation.

The direct vaccine effectiveness ${VE}_{i}$ after $i=0, 1,\ldots,n$ doses is defined^1^ as one minus the incidence rate (IR) ratio of vaccinated with exactly $i$ doses and non-vaccinated, indicated with $i=0$:

|  | ${VE}_{i}=1-\frac{{IR}_{i}}{{IR}_{0}} .$ | (S1) |
| --- | --- | --- |

The vaccine impact^3^ $VI$, also known as the overall vaccine effect^1^, is defined as one minus the incidence rate ratio between two populations, A and B:

|  | $VI=1-\frac{{IR}_{A}}{{IR}_{B}} .$ | (S2) |
| --- | --- | --- |

Population A is the population in which the immunization program is implemented. In general, depending on the uptake, such population consists of non-vaccinated, partially vaccinated and fully vaccinated persons. It follows that

|  | ${IR}_{A}=\sum_{i=0}^{n} x_{i}{IR}_{i},$ | (S3) |
| --- | --- | --- |

where $x_{i}$ is the proportion of the population that received $i$ doses. Population B is a control: subjects should be as similar as possible to population A, except that they are all non-vaccinated (in ecological studies, B is often the same population A before introducing the vaccine).^3^ Therefore,

|  | ${IR}_{B}={IR}_{0}$ | (S4) |
| --- | --- | --- |

Substituting formulas S3 and S4 in S2, using formula S2, we derive

|  | $VI=1- \sum_{i=0}^{n} x_{i}+\sum_{i=0}^{n} x_{i}{VE}_{i}.$ | (S5) |
| --- | --- | --- |

Since by definition $\sum_{i=0}^{n} x_{i}=1$, the first two terms cancel out, so that

|  | $VI=\sum_{i=1}^{n} x_{i}{VE}_{i},$ | (S6) |
| --- | --- | --- |

where the sum can start from $i=1$since the effectiveness of zero doses is trivially null, ${VE}_{0}=0$.

Therefore, the impact of a vaccination program with multiple doses is the sum of the VEs of each dose weighted by the proportions of population vaccinated with such number of doses. This simple formula holds in ideal situations in which both incidence rates and uptake proportions do not vary with age, time and other possible confounding factors. Also, it is valid only when indirect effects are small or absent. Otherwise additional factors could be needed in formulas S3 and S4, depending on the frequency of between-subject contacts that determine transmission.

The vaccination schedule in the 4CMenB national immunization program considered here was composed of three doses. Hence, formula S6 becomes:

|  | $VI=x_{1}{VE}_{1}+x_{2}{VE}_{2}+x_{3}{VE}_{3}.$ | (S7) |
| --- | --- | --- |

# S2 Cases data

Our data on cases were counts of serogroup B invasive meningococcal disease (IMD) in England, before and during the 4CMenB national immunization program, stratified by age group, year and doses received (Table S1). These data were provided by Public Health England.^4^

| **Table S1. Serogroup B IMD case counts shared by PHE and used for our re-assessment** |
| --- |
| \| **Age group** \| **Doses received** \| **Year of surveillance (September–August)** \| \| \| \| \| \| \| \| --- \| --- \| --- \| --- \| --- \| --- \| --- \| --- \| --- \| \| **2011-12** \| **2012-13** \| **2013-14** \| **2014-15** \| **2015-16** \| **2016-17** \| **2017-18** \| \| **0-1 m** \| **0** \| 14 \| 12 \| 19 \| 10 \| 7 \| 8 \| 10 \| \| **2-3 m** \| **0** \| 26 \| 20 \| 18 \| 18 \| 3 \| 8 \| 11 \| \| **1** \| - \| - \| - \| - \| 8 \| 13 \| 9 \| \| **2** \| - \| - \| - \| - \| - \| - \| - \| \| **3** \| - \| - \| - \| - \| - \| - \| - \| \| **4-11 m** \| **0** \| 113 \| 103 \| 86 \| 74 \| 36 \| 1 \| 5 \| \| **1** \| - \| - \| - \| - \| 13 \| 8 \| 10 \| \| **2** \| - \| - \| - \| - \| 9 \| 12 \| 13 \| \| **3** \| - \| - \| - \| - \| - \| - \| - \| \| **1 y** \| **0** \| 88 \| 77 \| 40 \| 80 \| 57 \| 13 \| 3 \| \| **1** \| - \| - \| - \| - \| 0 \| 0 \| 0 \| \| **2** \| - \| - \| - \| - \| 1 \| 7 \| 4 \| \| **3** \| - \| - \| - \| - \| 0 \| 9 \| 7 \| \| **2 y** \| **0** \| 49 \| 56 \| 28 \| 34 \| 42 \| 47 \| 7 \| \| **1** \| - \| - \| - \| - \| - \| 0 \| 0 \| \| **2** \| - \| - \| - \| - \| - \| 1 \| 2 \| \| **3** \| - \| - \| - \| - \| - \| 0 \| 10 \| \| **3 y** \| **0** \| 32 \| 27 \| 19 \| 21 \| 21 \| 24 \| 27 \| \| **1** \| - \| - \| - \| - \| - \| - \| 0 \| \| **2** \| - \| - \| - \| - \| - \| - \| 0 \| \| **3** \| - \| - \| - \| - \| - \| - \| 0 \| \| **4 y** \| **0** \| 29 \| 26 \| 18 \| 9 \| 22 \| 19 \| 21 \| \| **5-14 y** \| **0** \| 45 \| 60 \| 39 \| 48 \| 61 \| 46 \| 55 \| \| **15-24 y** \| **0** \| 79 \| 74 \| 63 \| 52 \| 75 \| 92 \| 94 \| \| **25-44 y** \| **0** \| 31 \| 33 \| 26 \| 19 \| 19 \| 27 \| 25 \| \| **45+ y** \| **0** \| 81 \| 73 \| 55 \| 56 \| 78 \| 72 \| 72 \| |
| *m: months; y: years.* |

# S3 Vaccine uptake and person-years data

We received data on 4CMenB’s uptake from Public Health England. We calculated the monthly proportions of the vaccinated population by month of age and number of doses from uptake statistics, following the same approach as in the original study analysis.^4^ First, we combined two datasets: i) monthly uptake data for each cohort reaching 6, 12 and 18 months of age, and ii) daily uptake data from nearly 60,000 individuals (30,000 for the booster dose) from different geographical areas across England. Then, uptake curves were shifted by 14 days to consider the time needed to develop an immune response. We considered 100% of the population born before May 2015 as non-vaccinated.

The annual number of person-years by age, stratified by number of vaccine doses injected, was derived by combining England’s population estimates with vaccine uptake. First, we downloaded England’s mid-year population estimates by year of age from the United Kingdom’s Office for National Statistics (<https://www.ons.gov.uk>). Then, we interpolated the data to mid-months (from September 2011 to August 2018) and month of age. After that, we used the above-described proportions of vaccinated individuals to further stratify England’s population by the number of vaccine doses received, from zero to three doses. Subsequently, we aggregated the population in the same age groups used for disease cases. Finally, we summed over months of time and divided by 12, to calculate the annual number of person-years for each age group and number of doses received. The resulting person-years are plotted in Figure S1 and Figure S2.

| **Figure S1. Person-years by age and year** |
| --- |
|  |
| *Total number of person-years by age group (horizontal axis) and year (September–August, different colors). We report here person-years independently of the number of doses they received (reported in figure S2).  m: months; y: years.* |

| Figure S2. Person-years / 100,000 by age, year and doses received |
| --- |
|  |
| *Person-years by age (from 0–1 m to 4 y, horizontal axes), year (2015–2018, columns) and number of doses received (0–3, rows). We report here only age groups and years for which the national immunization program was implemented. All the other age groups and years that are exclusively composed of non-vaccinated persons are reported in figure S1.  m: months; y: years.* |

# ­­­S4 Model’s inference

We used Bayesian inference to fit the IMD incidence model (equation 2 in the main manuscript), with non-informative prior distributions^5^ on $\rho\text{,} \theta\text{ and} \sigma_{\beta}$. We numerically derived posterior distributions using Markov chain Monte Carlo sampling with 40,000 iterations (four independent chains of size 10,000 after additional 5,000 burned tuning steps for each chain). Sampling was performed through the No-U-Turn sampler of Python’s PyMC3 package, a self-tuning variant of Hamiltonian Monte Carlo.^6–8^

Table S2 reports all the parameters.

| **Table S2. Best estimates of the parameters** |
| --- |
| \| **Parameter** \| **Posterior mean [95% BCI]** \| \| --- \| --- \| \| $\rho_{\text{0–1m}}$ \| -9.20 [-9.42; -8.98] \| \| $\rho_{\text{2–3m}}$ \| -8.58 [-8.76; -8.40] \| \| $\rho_{\text{4–11m}}$ \| -8.48 [-8.58; -8.38] \| \| $\rho_{\text{1y}}$ \| -9.20 [-9.30; -9.09] \| \| $\rho_{\text{2y}}$ \| -9.67 [-9.79; -9.55] \| \| $\rho_{\text{3y}}$ \| -10.24 [-10.40; -10.09] \| \| $\rho_{\text{4y}}$ \| -10.43 [-10.59; -10.26] \| \| $\rho_{\text{5-14y}}$ \| -11.75 [-11.86; -11.65] \| \| $\rho_{\text{15-24y}}$ \| -11.41 [-11.50; -11.33] \| \| $\rho_{\text{25-44y}}$ \| -13.26 [-13.41; -13.11] \| \| $\rho_{\text{≥45y}}$ \| -12.74 [-12.83; -12.65] \| \| $\theta_{1}$ \| -0.409 [-0.687; -0.132] \| \| $\theta_{2}$ \| -1.549 [-1.861; -1.257] \| \| $\theta_{3}$ \| -1.616 [-2.020; -1.214] \| \| $\sigma_{\beta}$ \| 0.151 [0.063; 0.266] \| |
| *The model and its parameters*$\rho\text{,} \theta\text{,} \sigma_{\beta}$ *are defined in the main manuscript (methods section). BCI: Bayesian credible intervals; m: months; y: years.* |

# S5 Model’s predictions

We sampled from posterior distributions (100,000 iterations) to generate posterior predictive distributions for the model’s best fitting parameters and expected cases.

Parameters’ posterior predictive distributions were used to calculate P values. For example, the probability that the VE after one dose is not higher than zero was calculated as the fraction of samples for which $\theta_{1}\geq0$ (i.e., for which ${VE}_{1}\leq0$, according to equation 3 of the main text).

Predictions of the number of expected cases for the no-vaccination scenario (the counterfactual, namely $Z$) were sampled in the same manner as the expected cases $Y$, except that all the effectiveness parameters were fixed to zero. Disease burden reduction imputable to the vaccine was calculated by subtracting the two predictive distributions as $Z- Y$.

In Figure S3 we compare observed data with expected cases. The data (i.e., disease case counts, also reported in table S1, shown here as black points) are stratified by age, time and number of doses received, and were reported in England between September 2011 and August 2018. Blue lines represent expected case counts from the best fit. Red lines represent expected counterfactual case counts that could have emerged if no vaccination program was implemented.

| Figure S3. Case data and model predictions by age, time and doses received |
| --- |
| 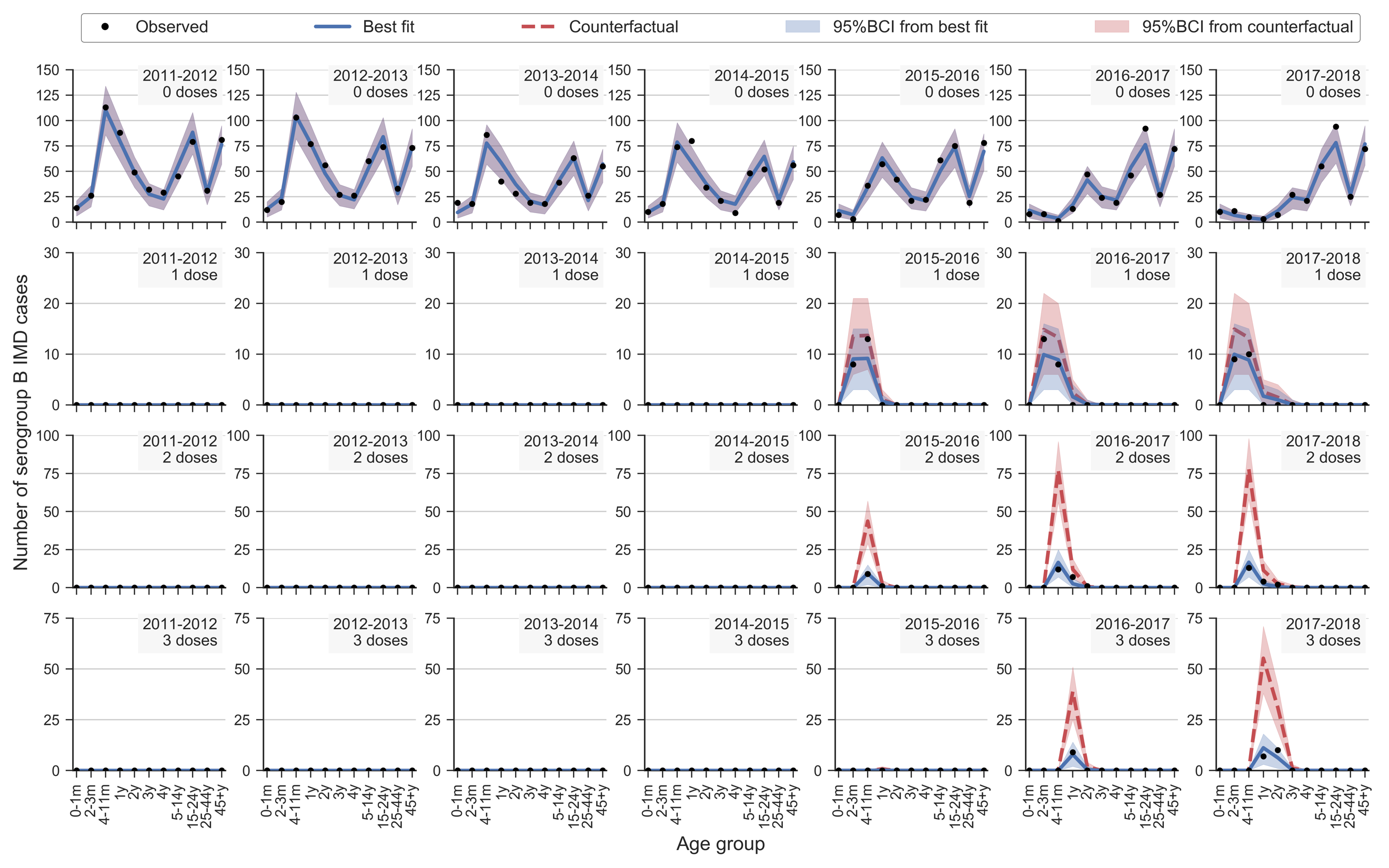 |
| *Each plot reports as black points the yearly number of observed serogroup B IMD case counts by age for a different year and number of doses received (from zero to three). Expected case counts from incidence model’s best fit are shown as blue lines. Counterfactual case counts, generated through the same model when setting all the VE parameters to zero, are shown as red dashed lines. The blue and red semi-transparent regions are the 95% BCI of the corresponding curves.  BCI: Bayesian credible intervals; m: months; y: years.* |

# S6 Predictive accuracy of the incidence model

We quantified the predictive accuracy of the best fitting incidence model (equation 2 in the main text, also called P1 model) for expected *vs.* observed cases using the Bayesian R-squared^9^ (variance of the predicted values divided by the variance of predicted values plus the expected variance of the errors):

$R^{2}=0.963$.

Figure S4 shows residuals versus observed cases (panel A) and predicted cases vs. observed cases (panel B).

| Figure S4. Residuals and predicted vs. observed cases | |
| --- | --- |
| A |  |
| B |  |

# S7 Alternative models

## P2 model

$$y_{a,t,d} \sim Poisson \left( N_{a,t,d}e^{\rho_{a}+\beta_{t}+\theta_{d}} \right)$$

Compared to the P1 model (equation 2 in the main manuscript), the $\beta_{t}$ parameter is not constrained to follow a normal distribution (therefore, the effective number of parameters is greater than in P1). Therefore, all seven $\beta_{t}$ parameters are independently generated rather than hierarchically derived from a Gaussian process of mean zero and variance $\sigma_{\beta}^{2}$ (second line in equation 2 of the main manuscript).

## P3 model

$$y_{a,t,d} \sim Poisson \left( N_{a,t,d}e^{\rho_{a}+\theta_{d}} \right)$$

Compared to the P1 model (equation 2 in the main manuscript), the $\beta_{t}$ parameter is fixed to zero, i.e., there is no adjustment for time variations of incidence.

## P4 and P5 model

$$y_{a,t,d} \sim Poisson \left( N_{a,t,d}e^{\gamma_{a,t}+\theta_{d}} \right)$$

Compared to the P1 model (equation 2 in the main manuscript), the $\rho_{a}+\beta_{t}$ parameter is replaced by $\gamma_{a,t}$, which depends on both age and time. Therefore, the P4 and P5 models do not assume independence between age and time, at the price of a greater number of parameters (with 11 age groups and 7 time points, it is 11x7 = 77 parameters for $\gamma_{a,t}$, compared to 11+7 = 18 parameters for $\rho_{a}+\beta_{t}$). P4 is hierarchical ($\gamma_{a,t}$ is constrained to follow a normal distribution of mean zero and variance $\sigma_{\gamma}^{2}$); thus, the effective number of parameters is actually lower than for P5, for which all the $\gamma_{a,t}$ parameters are fully independent.

## NB models

Negative binomial models NB1, NB2, NB3, NB4 and NB5 correspond to Poisson models $\mathrm{Poisson}\left( \mu\right)$ for which rate parameters $\mu$ follow a gamma distribution. Negative binomial distributions are used here as an extension of the Poisson count model. Having an additional parameter, NB can account for overdispersion when the variance of the counts is greater than the mean. NB models have been parametrized in terms of $\mu>0$, corresponding to the above-defined P1, P2, P3, P4 and P5 models, with the additional parameter $\alpha>0$ that models overdispersion:

$$y_{a,t,d} \sim NegBin \left( \mu_{a,t,d}, \alpha\right)$$

For a large $\alpha$, the negative binomial distribution $\mathrm{NegBin}\left( \mu, \alpha\right)$ approaches a Poisson distribution $\mathrm{Poisson}\left( \mu\right)$ of mean $\mu$.

Having defined NB models as the negative binomial counterparts of P models, the specific mathematical expression for $\mu_{a,t,d}$ can be found in the respective P models. For example, the NB1 model is

$y_{a,t,d} \sim NegBin \left( N_{a,t,d}e^{\rho_{a}+\beta_{t}+\theta_{d}}, \alpha\right)$,

with a hierarchically distributed $\beta_{t}$

$\beta_{t} \sim Normal \left( 0; \sigma_{\beta}^{2} \right)$.

# S8 Convergence diagnostics and posterior distributions of sampled parameters for the best fitting model

| **Figure S5. Sampled parameters and their distributions** | |
| --- | --- |
| 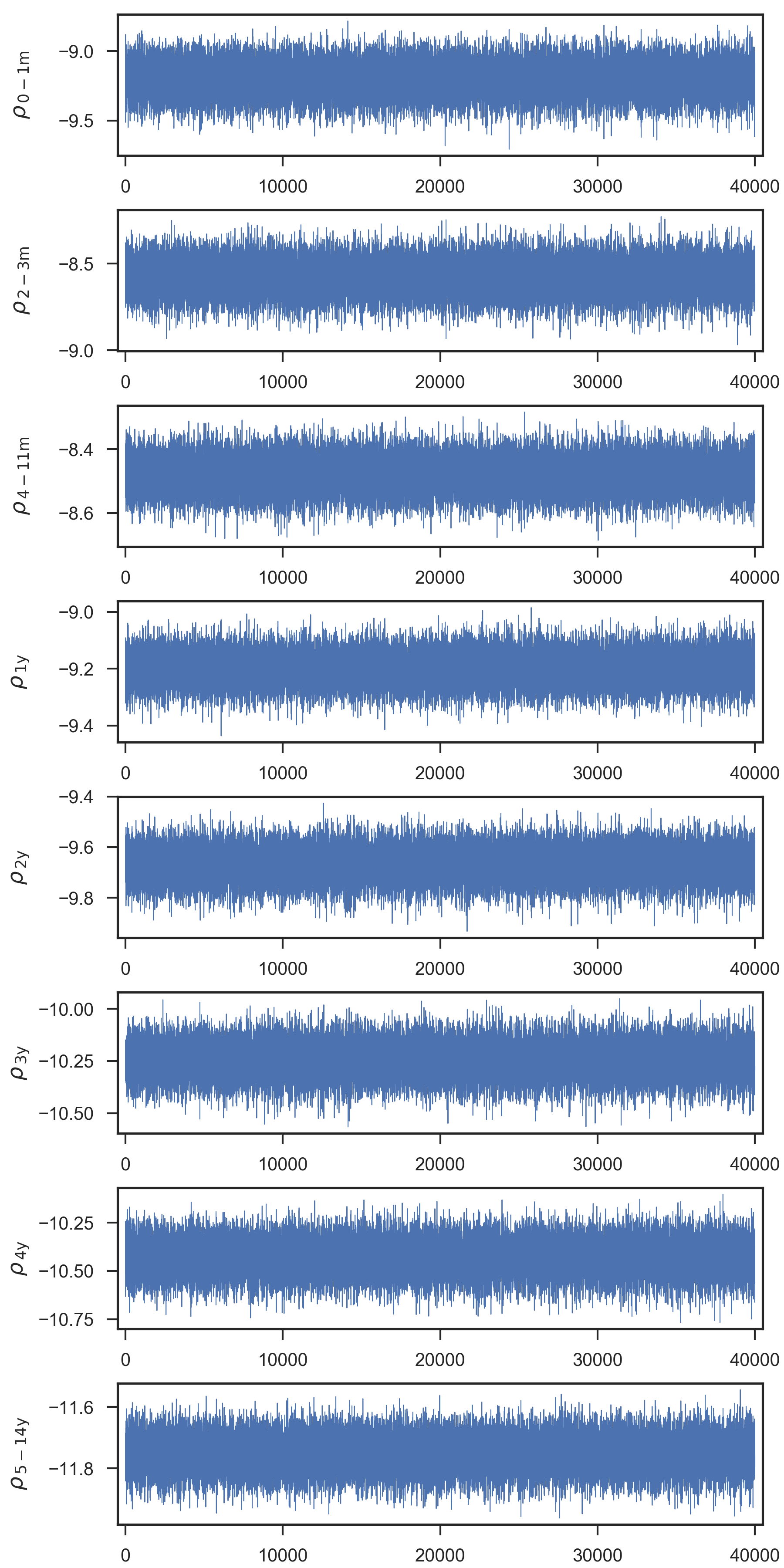 | 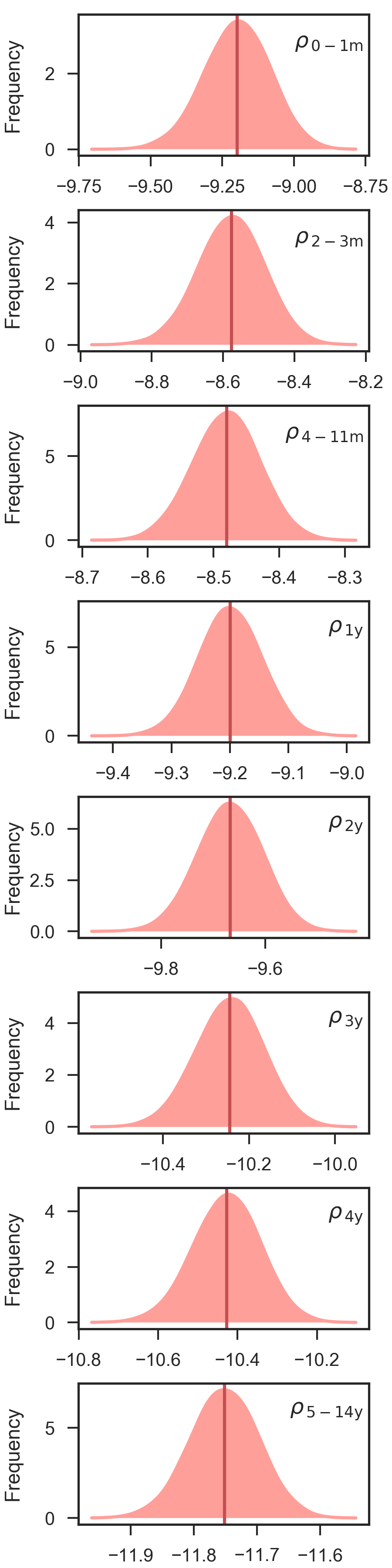 |
| Continued on next page. | |

| **Figure S5. continued** | |
| --- | --- |
| 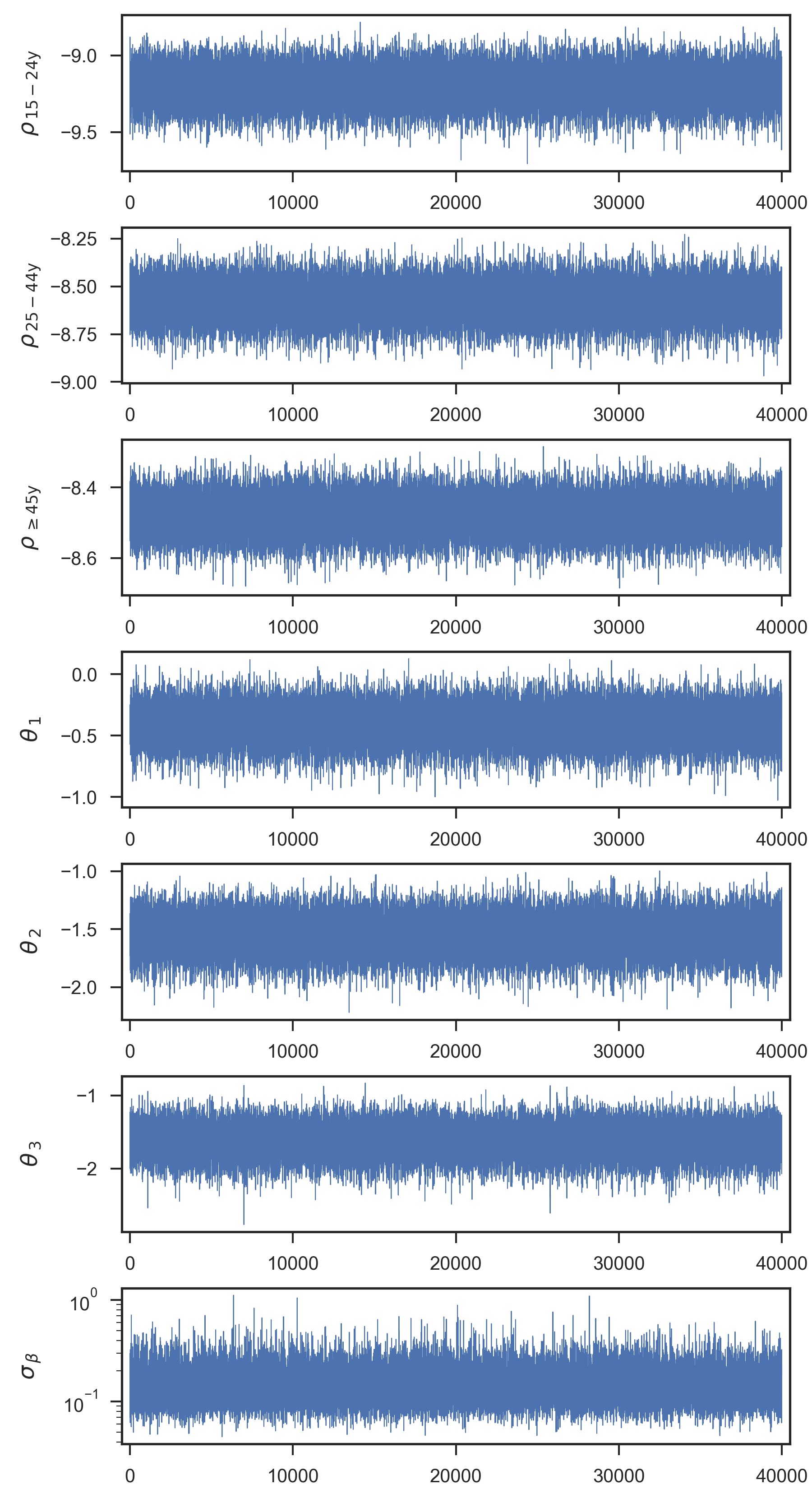 | 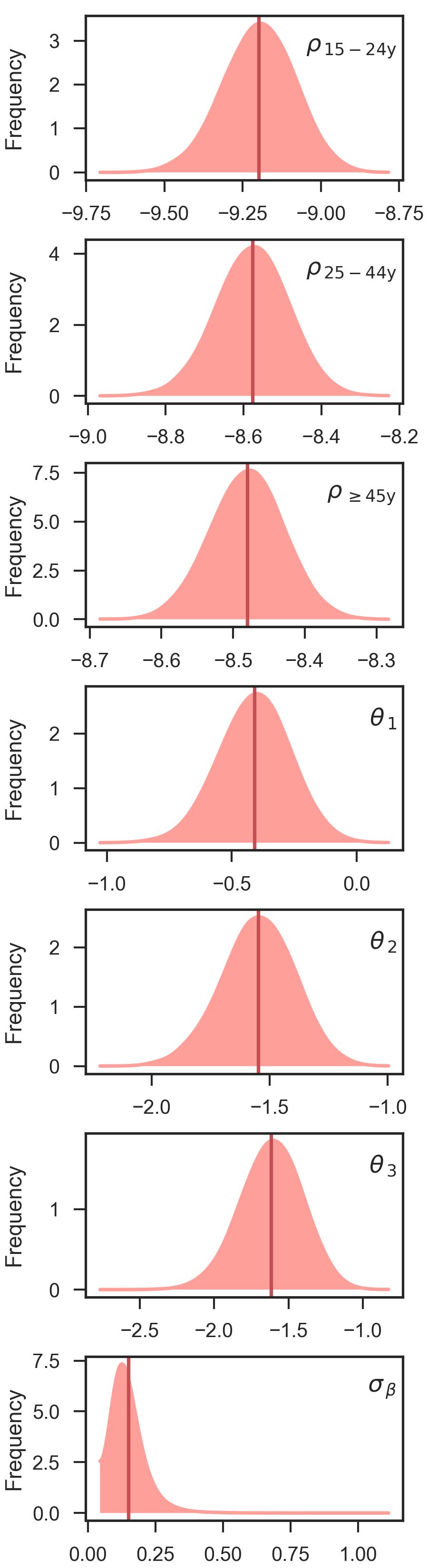 |
| *For each parameter of the best fitting P1 model (equation 2 of the main manuscript) this figure displays, on the left side, the sampled traces (40,000 iterations). The traces show that the Monte Carlo algorithm converged well. On the right side, each figure displays the respective posterior distributions of the sampled parameters (parameter values shown on horizontal axes), together with their means shown as vertical lines (specific values reported in table S2).* | |

References

1. Halloran ME, Longini Jr. IM, Struchiner CJ. *Design and Analysis of Vaccine Studies: Introduction*. Springer; 2009. http://link.springer.com/10.1007/978-0-387-68636-3.

2. Amirthalingam G, Andrews N, Keel P, et al. Evaluation of the effect of the herpes zoster vaccination programme 3 years after its introduction in England: a population-based study. *Lancet Public Heal*. 2018;3(2):e82-e90. doi:10.1016/S2468-2667(17)30234-7

3. Hanquet G, Valenciano M, Simondon F, Moren A. Vaccine effects and impact of vaccination programmes in post-licensure studies. *Vaccine*. 2013;31(48):5634-5642. doi:10.1016/j.vaccine.2013.07.006

4. Ladhani SN, Andrews N, Parikh SR, et al. Vaccination of infants with meningococcal group B vaccine (4CMenB) in England. *N Engl J Med*. 2020;382(4):309-317. doi:10.1056/NEJMoa1901229

5. Gelman A, Carlin JB, Stern HS, Dunson DB, Vehtari A, Rubin DB. *Bayesian Data Analysis, Third Edition*. Taylor & Francis; 2013. https://books.google.it/books?id=ZXL6AQAAQBAJ.

6. Salvatier J, Wiecki T V., Fonnesbeck C. Probabilistic programming in Python using PyMC3. *PeerJ Comput Sci*. 2016;2:e55. doi:10.7717/peerj-cs.55

7. Hoffman MD, Gelman A. The no-U-turn sampler: Adaptively setting path lengths in Hamiltonian Monte Carlo. *J Mach Learn Res*. 2014;15:1593-1623.

8. Duane S, Kennedy AD, Pendleton BJ, Roweth D. Hybrid Monte Carlo. *Phys Lett B*. 1987;195(2):216-222. doi:10.1016/0370-2693(87)91197-X

9. Gelman A, Goodrich B, Gabry J, Vehtari A. R-squared for Bayesian Regression Models. *Am Stat*. 2019;73(3):307-309. doi:10.1080/00031305.2018.1549100
